# Is Metabolic Syndrome Truly a Risk Factor for Male Lower Urinary Tract Symptoms or Just an Epiphenomenon?

**DOI:** 10.1155/2014/203854

**Published:** 2014-01-23

**Authors:** Marina Zamuner, Walker Wendell Laranja, João Carlos Cardoso Alonso, Fabiano A. Simões, Ronald F. Rejowski, Leonardo O. Reis

**Affiliations:** ^1^Faculty of Medicine, Pontifical Catholic University of Campinas (PUC-Campinas), 13060-904 Campinas, SP, Brazil; ^2^Paulínia Municipal Hospital, 13140-295 Paulínia, SP, Brazil; ^3^Faculty of Medicine and Urology Division, University of Campinas (Unicamp), 13083-887 Campinas, SP, Brazil

## Abstract

To define whether the association of male lower urinary tract symptoms (LUTS) and metabolic syndrome (MS) is real or simply an epiphenomenon, 490 male adults (mean age 58 ± 9 years) underwent International Prostate Symptom Score (IPSS), physical and prostate digital examinations, blood analysis, and urinary tract transabdominal ultrasound with prostate volume measurement. Mild, moderate, and severe LUTS were found in 350 (71.4%), 116 (23.7%), and 24 (4.9%) patients, respectively. MS was present in 198 (40.4%) patients, representing 37.4% (131 of 350) of those with mild LUTS, 46.5% (54 of 116) of those with moderate, and 54.1% (13 of 24) of those with severe. The odds ratio of MS having moderate or severe LUTS was 2.1. MS was more common in older age, higher body mass index, and larger prostate size. Moderate and severe LUTS were more frequent in older age, lower levels of high density cholesterol, and higher blood pressure. Older age and body mass index had significant relative risk for lower urinary tract symptoms and only age remained independent factor for LUTS on multivariate analysis. Our results suggest that the association of male LUTS, prostate volume, and MS might be coincidental and related to older age.

## 1. Introduction

Lower urinary tract symptoms (LUTS) in old men are generally attributed to benign prostatic enlargement (BPE) and may be associated with metabolic syndrome (MS) [1] due to several putative hormonal pathways that may lead to both conditions [2]. BPE and MS are highly prevalent diseases especially in the aged population [3, 4] leading to doubt whether the association of these conditions is real or simply an epiphenomenon.

This study aims to correlate male lower urinary tract symptoms, prostate volume, and metabolic syndrome.

## 2. Methods

### 2.1. Population

After exclusion of patients with previous prostate or urethral operations (*n* = 11), we studied 490 unselected and consecutive male adults, of mean age 58 ± 9 (36–84) years, from an institutional review board approved prospectively kept database originated from a community hospital general urologic clinic in a cross-sectional study.

All individuals were routinely questioned, at the time of the first consultation, in regards to lower urinary tract symptoms, and had a full physical examination, prostate digital rectal examination, blood collected for laboratory analysis, and a baseline urinary tract transabdominal ultrasound with prostate volume measurement.

### 2.2. Male Lower Urinary Tract Symptoms

Male LUTS were defined based on the International Prostate Symptom Score (IPSS). The score ranges from 0 to 35. A score of 0–7 is defined as mildly symptomatic, 8–19 as moderately symptomatic, and 20–35 as severely symptomatic [5].

### 2.3. Metabolic Syndrome

MS was defined based on the US National Cholesterol Education Program Adult Treatment Panel III, 2001 [6].

### 2.4. Statistics

Variables are expressed as mean ± standard deviation (range).

Student's *t*-test, odds ratio (confidence interval), relative risk (confidence interval), and logistic regression were used when indicated. Multivariate analysis (bias-reduced logistic regression) considered presence of mild versus moderate + severe LUTS as the dependent variable. Statistical power was calculated comparing the proportions of LUTS and MS with a tolerated alpha error level of 5%.

The two-sided value of *P* < 0.05 was considered significant.

## 3. Results

Mild, moderate, and severe LUTS were found in 350 (71.4%), 116 (23.7%), and 24 (4.9%) patients, respectively. Overall, MS was present in 198 (40.4%) patients, representing 37.4% (131 of 350) of those with mild LUTS, 46.5% (54 of 116) of those with moderate, and 54.1% (13 of 24) of those with severe LUTS.

The odds ratio of patients with MS having moderate or severe LUTS was 2.1 (95% CI = 1.41 to 3.13) with a statistical power of 98.6%.

Table 1 shows demographic and prostatic parameters of patients according to the presence of metabolic syndrome. MS was more common in older age, higher body mass index, and larger prostate size.

Table 2 shows demographic and metabolic parameters of patients according to lower urinary tract symptoms. Moderate and severe LUTS were more frequent in older age, lower levels of high density cholesterol, higher blood pressure, and higher prostate volume.

Table 3 shows the relative risk for lower urinary tract symptoms based on metabolic parameters. Only older age and higher body mass index were significant risks for lower urinary tract symptoms.

Only age remained as an independent factor for lower urinary tract symptoms after multivariate analysis (Table 4).

## 4. Discussion

Considering the potential for preventive public health attitudes, it is important to predict the events leading to LUTS and BPE progression and recent studies have suggested a possible association between the metabolic syndrome and the occurrence of LUTS/BPE, with possible new targets for prevention and treatment of these disorders, although the evidence for a causal relationship remains missing [7].

Our results show that (1) MS brings a 2-fold risk for male lower urinary tract symptoms, (2) older age and body mass index are risk factors for male lower urinary tract symptoms, and (3) older age, lower levels of high density cholesterol, and higher blood pressure are risk factors for moderate and severe LUTS; however, only age remained as an independent factor for male lower urinary tract symptoms on multivariate analyses.

### 4.1. Correlation among Male Lower Urinary Tract Symptoms, MS, Prostate Volume, and Age

The correlation between male lower urinary tract symptoms and MS is a controversial topic. While some authors advocate a link between the diseases [8, 9] others have not found this association significant [10–13]. LUTS in old men are generally attributed to BPE [1] and BPE may be related to MS [14]. In fact, our results show that MS is more common in patients with larger prostates, even though benign prostatic hyperplasia (BPH) was not confirmed by biopsy. Thus, we have limited our evaluation to BPE, a clinical entity representing the pathological entity BPH. Age may be the essential mutual factor between MS and LUTS since both conditions are prevalent in the elderly population. Severe LUTS may be found in 10% of males older than 70 years [15] and 52% of males 60 years and over will have MS in the United States [16].

Our results showed some risk factors simultaneously for MS and male lower urinary tract symptoms. However, older age is also a common feature for these putative risk factors. Higher body mass index has been described as a common pathway between male lower urinary tract symptoms and MS [17]. The prevalence of obesity increases with age [18]. Similarly, low levels of high density cholesterol are common in the elderly [19] and a mutual characteristic of MS and male lower urinary tract symptoms [20]. Finally, high blood pressure shares the same characteristic of high prevalence with advanced age [19]; this also happens in male patients with lower urinary tract symptoms and MS [21].

After adjusting for age and testosterone, there was no association between MS and male lower urinary tract symptoms measured by the previously validated tool IPSS in our series of Latin American patients. To add controversy to the issue, metabolic syndrome was also previously described as having favorable effects on male lower urinary tract symptoms [9, 22]. Compared to the non-MS group, men in the MS group were less likely to experience moderate to severe LUTS elsewhere (OR 0.58, 95% CI 0.41–0.83) [9].

### 4.2. Study Limitations

Our study has some potential limitations. First, this is a retrospective case series from a community hospital; nonetheless, a significant number of patients were included in the study bringing an effective statistic power. Also, the prevalence of MS in Brazil is high [23], 40.4% in our study, favoring the proposed comparison between MS and male lower urinary tract symptoms. Second, prostate biopsy was not available for most patients to allow the precise diagnosis of BPH in order to correlate LUTS and BPH; alternatively we focused on prostate volume or BPE, which represents virtually all BPH. Additionally, the previously validated IPSS (male LUTS) is a more reliable and precise tool, figuring as a key outcome on the subject considering its accuracy to determine the main impact of BPH or causative factors on patients' quality of life, which is what really matters.

Finally, considering the debatable literature on the issue [8–10, 13, 14, 22] and the fact that most studies to date come from Asia where metabolic syndrome incidence is as low as 10–20% compared to over 40% in the present study, report of every series is important to add knowledge to this controversial subject, especially a Latin American series.

## 5. Conclusions

Our results show that the association of male lower urinary tract symptoms, prostate volume, and MS may be coincidental and related to older age.

## Figures and Tables

**Table 1:** Demographic parameters of patients according to the presence of metabolic syndrome.

Continuous variables	Metabolic syndrome (*n* = 198)	Nonmetabolic syndrome (*n* = 292)	*P *
Age (years)	59 ± 9	57 ± 9	**0.02**
Body mass index (Kg/m^2^)	30 ± 4	26 ± 4	**<0.001**
Prostate volume (cm^3^)	31 ± 9	29 ± 8	**0.01**
Total PSA	1.69 ± 4.1	1.49 ± 4.0	0.30
Total testosterone (ng/dL)	486 ± 250	512 ± 251	0.26
Free testosterone (ng/dL)	90 ± 66	97 ± 67	0.25
IPSS	5 ± 7	7 ± 7	0.12

PSA: prostate specific antigen; IPSS: International Prostatic Symptoms Score.

Data in bold font refers to *P* value <0.05.

**Table 2:** Demographic and metabolic parameters of patients according to lower urinary tract symptoms (IPSS).

Continuous variables	Mild (*n* = 350)	Moderate (*n* = 116)	Severe (*n* = 24)	*P *
Age (years)	56 ± 8	61 ± 8	63 ± 9	Mild versus moderate **<0.001**
				Mild versus severe **<0.001**
				Moderate versus severe = 0.3

Body mass index (Kg/m^2^)	28 ± 5	28 ± 5	28 ± 4	1

Abdominal circumference (cm)	98 ± 10	100 ± 11	97 ± 11	Mild versus moderate 0.07
				Mild versus severe 0.6
				Moderate versus severe 0.2

Triglycerides (mg/dL)	153 ± 104	152 ± 89	117 ± 62	Mild versus moderate 0.9
				Mild versus severe 0.09
				Moderate versus severe 0.07

Cholesterol (mg/dL)	193 ± 40	192 ± 39	184 ± 31	Mild versus moderate 0.8
				Mild versus severe 0.3
				Moderate versus severe 0.3

High density cholesterol (mg/dL)	44 ± 15	40 ± 12	37 ± 15	Mild versus moderate **0.01**
				Mild versus severe = **0.03**
				Moderate versus severe 0.3

Glycemia (mg/dL)	108 ± 37	113 ± 41	103 ± 26	Mild versus moderate 0.2
				Mild versus severe 0.5
				Moderate versus severe 0.2

Systolic blood pressure (cm Hg)	12 ± 1	13 ± 1	13 ± 1	Mild versus moderate **<0.001**
				Mild versus severe **<0.001**
				Moderate versus severe 1

Diastolic blood pressure (cm Hg)	8 ± 1	8 ± 1	9 ± 1	Mild versus moderate 1
				Mild versus severe **<0.001**
				Moderate versus severe **<0.001**

Prostate volume (cm^3^)	29 ± 9	33 ± 9	33 ± 10	Mild versus moderate **<0.001**
				Mild versus severe **0.04**
				Moderate versus severe 1

Data in bold font refers to *P* value <0.05.

**Table 3:** Relative risk for lower urinary tract symptoms based on demographic and metabolic parameters.

Categorical variables	LUTS mild (*n* = 350)	LUTS moderate/severe (*n* = 140)	Relative risk (95% CI)
Age > 60 years	125 (36%)	133 (95%)	17.08 (8.16 to 35.78)
BMI > 30 kg/m^2^	91 (26%)	137 (98%)	52.48 (16.95 to 162.46)
AC > 102 cm	91 (26%)	40 (28%)	1.10 (0.81 to 1.49)
TG > 150 mg/dL	137 (39%)	47 (33%)	0.84 (0.62 to 1.13)
HDL < 40 mg/dL	141 (40%)	74 (53%)	1.43 (1.08 to 1.90)
Gly > 110 mg/dL	88 (25%)	39 (28%)	1.10 (0.81 to 1.50)
SBP > 13 cm Hg	136 (39%)	80 (57%)	1.69 (1.27 to 2.24)
DBP > 8 cm Hg	107 (30%)	56 (40%)	1.34 (1.00 to 1.77)
Prostate > 40 cm^3^	89 (25%)	50 (36%)	1.40 (1.05 to 1.86)

LUTS: lower urinary tract symptoms; BMI: body mass index; AC: abdominal circumference; TG: triglycerides; HDL: high density cholesterol; Gly: glycemia; SBP: systolic blood pressure; DBP: diastolic blood pressure.

**Table 4:** Multivariate analysis for the presence of mild versus moderate + severe male LUTS as the dependent variable.

Variables	*P* value
Age	**<0.001**
Body mass index	0.4
Total cholesterol	0.4
High density cholesterol	0.4
Low density cholesterol	0.2
Triglycerides	0.7
Glycemia	0.7
Systolic blood pressure	0.1
Diastolic blood pressure	0.7
Abdominal circumference	0.3

Data in bold font refers to *P* value <0.05.
